# Ultrathin graphene oxide-based hollow fiber membranes with brush-like CO_2_-philic agent for highly efficient CO_2_ capture

**DOI:** 10.1038/s41467-017-02318-1

**Published:** 2017-12-13

**Authors:** Fanglei Zhou, Huynh Ngoc Tien, Weiwei L. Xu, Jung-Tsai Chen, Qiuli Liu, Ethan Hicks, Mahdi Fathizadeh, Shiguang Li, Miao Yu

**Affiliations:** 1 Department of Chemical & Biological Engineering, Rensselaer Polytechnic Institute, Troy, NY 12180 USA; 2 Department of Chemical Engineering and Catalysis for Renewable Fuels Center, University of South Carolina, Columbia, SC 29208 USA; 3 Gas Technology Institute, 1700 S Mount Prospect Road, Des Plaines, IL 60018 USA

## Abstract

Among the current CO_2_ capture technologies, membrane gas separation has many inherent advantages over other conventional techniques. However, fabricating gas separation membranes with both high CO_2_ permeance and high CO_2_/N_2_ selectivity, especially under wet conditions, is a challenge. In this study, sub-20-nm thick, layered graphene oxide (GO)-based hollow fiber membranes with grafted, brush-like CO_2_-philic agent alternating between GO layers are prepared by a facile coating process for highly efficient CO_2_/N_2_ separation under wet conditions. Piperazine, as an effective CO_2_-philic agent, is introduced as a carrier-brush into the GO nanochannels with chemical bonding. The membrane exhibits excellent separation performance under simulated flue gas conditions with CO_2_ permeance of 1,020 GPU and CO_2_/N_2_ selectivity as high as 680, demonstrating its potential for CO_2_ capture from flue gas. We expect this GO-based membrane structure combined with the facile coating process to facilitate the development of ultrathin GO-based membranes for CO_2_ capture.

## Abstract (web summary)

Membrane separation technologies show promise for CO_2_ capture, but typically suffer from a trade-off between permeance and selectivity. Here, the authors produce hollow fiber membranes coated with graphene oxide and a CO_2_-philic agent that can efficiently separate CO_2_ from flue gas under wet conditions.

## Introduction

Emission of CO_2_, arising from the combustion of fossil fuels, has been considered a primary reason for human-induced climate change and raised a widespread environmental concern^1^. The 2020 Carbon Capture Program, administered by the US Department of Energy (DOE), announced the post-combustion capture goal of achieving 90% CO_2_ capture with <35% increase in the levelized cost of energy^2^. To achieve this goal, various carbon capture technologies, such as adsorption, absorption, and membrane separation, have been widely investigated^3^. Among these technologies, membrane separation, which is based on the difference of gas permeation rates through membrane barriers, possesses many inherent advantages, such as high energy efficiency, low capital investments, operational simplicity, and flexibility to scale up, and thus has been considered as a very promising technology^4^.

Polymer membranes were first proposed for CO_2_ separation in the late 1970s due to their superior film-forming ability and processability^5^. Many following studies have been conducted to explore the potential of polymer membranes for CO_2_ separation^6–8^. In particular, MTR (Membrane Technology and Research, Inc.) reported a Polaris™ membrane with a CO_2_ permeance of 1000 to 2000 GPU (Gas Permeance Unit; 1GPU = 1 × 10^−6^ cm^3^(STP)/cm^2^ s cm Hg = 3.348 × 10^−10^ mol m^−2^ s^−1^ Pa^−1^) and a CO_2_/N_2_ mixture separation selectivity of around 25. This membrane has been scaled up to a 2000 m^2^ pilot scale system and tested for coal-fired flue gas CO_2_ capture at the National Carbon Capture Center^9^. Polymer membranes, however, due to the intrinsic mobility of polymer chains, suffer the trade-off between permeability and selectivity, and thus are usually constrained by an upper bound limit^2, 10, 11^. Non-polymer fillers, such as zeolites^12^, silicon^13^, metal organic framework (MOF)^14^, and carbon derivatives^15^, have been extensively added to the polymeric matrix to prepare mixed matrix membranes (MMM), and investigated for their improvement on membrane separation performance. However, it still remains a big challenge to accomplish highly permeable and selective membranes for CO_2_ capture^16^. New generation membranes having novel structures and/or composed of novel materials were then developed to greatly enhance the separation performance and stability^16^. For example, the Ohio State University developed a polymer-zeolite composite flat-sheet membrane with a three-layer novel structure, and achieved CO_2_ permeance of 800 GPU and CO_2_/N_2_ selectivity of 200 at 57 °C^17^.

Graphene oxide (GO), as a novel two-dimensional material, has been considered as a promising material for making GO-based separation membranes with GO as the dominant/skeleton membrane material and MMM membranes as an additive^18–24^. Kim et al.^19^. deposited ultrathin GO by spin-casting, and found a high CO_2_/N_2_ selectivity of around 50 in high relative humidity (85%). They speculated that the highly interlocked GO-layered structure and the condensed water molecules in the pores or between layers hindered the transport of non-condensable small gas molecules but not CO_2_, and thus led to gas selectivity. In contrast, our group^20^ fabricated ultrathin GO membranes by vacuum filtration, and demonstrated excellent H_2_/CO_2_ separation performance, probably through structural defects on GO flakes. Recently, Wang et al.^21^ prepared a borate-crosslinked GO membrane via vacuum filtration, and a relatively high CO_2_ permeance (650 GPU) was obtained with CO_2_/N_2_ selectivity of around 50 at 30 °C. The tuned GO interlayer nanochannel size endowed the membrane with size sieving ability for different gas molecules. In the meantime, much work using GO as additive in polymer membrane (MMM) has also been performed for gas separation^22–25^, but usually low gas permeance and selectivity were found.

To date, however, only a few studies^19, 21, 26^ investigated CO_2_ capture from flue gas using GO-based membranes as discussed above, and these studies commonly employed spin-casting and vacuum filtration methods for GO deposition on flat-sheet supports. Relatively low CO_2_ permeance and/or selectivity was reported, and these current GO deposition methods are difficult to scale up, especially on hollow fibers, which have relatively high packing density, compared with flat sheet supports. Therefore, there is a strong need to design more effective GO-based membrane structure and develop scalable GO deposition method to further explore their potential for CO_2_ capture. Herein, we report a layered GO-based membrane with brush-like CO_2_-philic agent alternating between layers prepared by a facile and potentially scalable coating method for hollow fibers. To the best of our knowledge, this is the first reporting of GO-based membranes on the inner surfaces of polymeric hollow fiber supports for gas separation purposes; GO coating on the inner surface may be beneficial for membrane protection. Moreover, owing to the novel brush-like, CO_2_-philic agent grafted between GO layers, the membranes show superior separation performance to other membranes reported in the literature. We expect the structure of the GO-based membrane with replaceable CO_2_-philic agent and facile GO deposition method would build a strong platform for further developing GO-based membranes for highly effective CO_2_ capture.

## Results

### Membrane structure and fabrication process

**Fig. 1:**
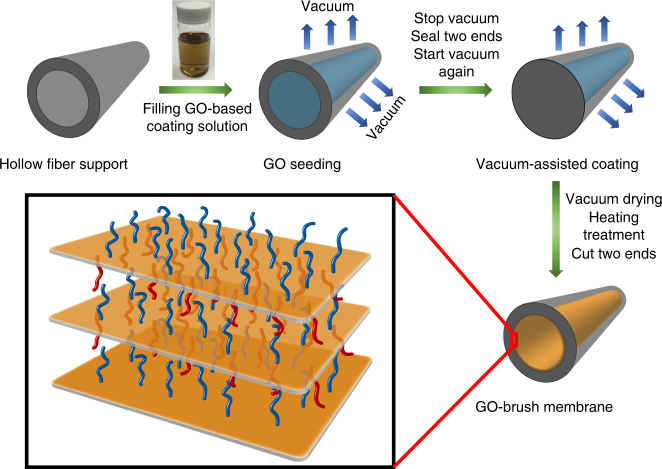
Fabrication process and structure diagram of the ultrathin GO-based hollow fiber membranes with brush-like CO_2_-philic agent. The blue brushes denote the grafted agents on the top surface of GO, and the red brushes denote the grafted agents on the bottom surface of GO. The brushes can be appropriate CO_2_-philic agents that are able to chemically bond with the GO surface by reacting with oxygen-functional groups

We developed a process, as shown in Fig. 1, to uniformly deposit the GO-based membranes on the inner surface of a commercial polymeric hollow fiber support via a two-step modified vacuum-assisted filtration using a home-designed hollow fiber coating system (Supplementary Fig. 1). GO was synthesized by modified Hummers method^27^, and then dispersed in water as coating solution. Atomic force microscopy (AFM) image (Supplementary Fig. 2 **)** showed the individual GO flakes, and the depth profile indicated that GO sheet is single-layered graphene oxide (SLGO) with thickness of about 1 nm. For GO deposition on hollow fiber in this work, different coating processes based on vacuum filtration were conducted in an attempt to deposit a high quality and selective GO coating layer on the inner surface of hollow fibers (see Supplementary Table 1). However, most of these processes resulted in membranes with no apparent CO_2_/N_2_ selectivity and similar gas permeance as that through the support for both gases. We then established a novel GO seeding and modified vacuum-assisted coating process, and successfully deposited ultrathin GO coatings on hollow fiber support, which showed comparable CO_2_/N_2_ separation performance with that prepared by spin coating (See discussion). The inner channel of hollow fiber support was first filled with the coating solution completely using a syringe pump before applying vacuum. The GO seeding process immediately began when the vacuum was applied from outside of the support. SLGO sheets are expected to rapidly pre-deposit on the polyethersulfone (PES) hollow fiber inner surface to improve the surface hydrophilicity and reduce the support surface roughness. Note that during the GO seeding process, the PES hollow fiber was completely filled with solution at all times by keeping the entrance and exit of the hollow fiber open. We found that the seeding step is critical to ensure high-quality GO coating, and without this step poor coating layer with no apparent CO_2_/N_2_ selectivity always resulted. After seeding, the entrance and exit of the hollow fiber were sealed, and then vacuum was applied again to completely remove water and form a high quality and uniform coating layer. Detailed coating procedure is presented in the Supplementary Information (see Supplementary Methods). Individual hollow fibers were used for coating process in this study.

### Membrane performance enhancement by addition of CO_2_-philic agents

Compared with other two-dimensional materials, GO has inherent structural flexibility^28^. This allows it for effective assembly of 2-D nanostructures with appropriate additives for different purposes. In this work, a layered GO membrane structure with brush-like, CO_2_-philic agent alternating between GO layers was designed and fabricated by chemically introducing a CO_2_ carrier into a few-layered GO nanochannels. As reported in previous studies^2, 21, 29^, CO_2_ carriers in facilitated transport membranes considerably increase CO_2_ permeance. Among all the CO_2_-philic agents, amines, as the most mature and highly efficient CO_2_ absorbents, have been well developed since last century^30^. Piperazine was selected in this work because it is the most effective amine promoter for CO_2_ capture due to its rapid formation of carbamate with CO_2_^30, 31^. We effectively incorporated piperazine-brushes into GO nanochannels to form a new structure by the developed coating method to achieve highly permeable and highly selective layered GO-piperazine (GOP) hollow fiber membranes. During membrane preparation, GO suspension was simply replaced by the GO suspension with dissolved piperazine, and the coating process was the same as GO membrane preparation. The as-coated GOP hollow fiber membranes were then heated at 80 °C in an oven for 1 h to form a high degree of chemical bonding between GO and piperazine molecules. Note that the melting point of piperazine is 106 °C. This ensures long reaction time between piperazine and the GO surface functional groups during heating and thus maximizes chemical bonding between them. The brushed and alternated structure of the GOP layer is shown in Fig. 1.

### Membrane morphology and composition characterization

**Fig. 2:**
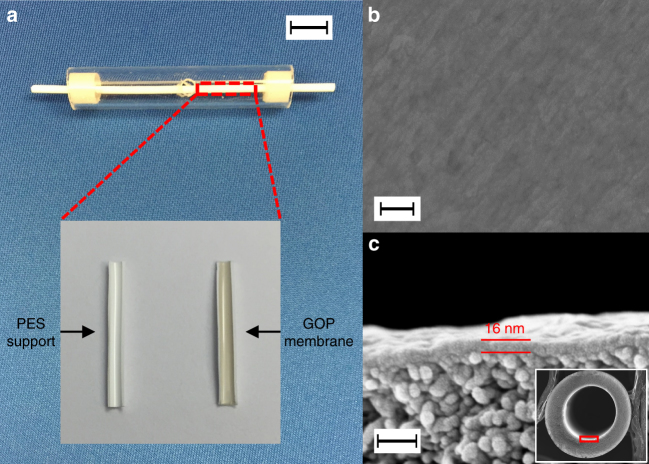
GO-piperazine hollow fiber membranes. **a** Digital picture of a GO-piperazine (GOP) hollow fiber membrane housed in a Pyrex tube. The scale bar is 10 mm; inset shows the inner surface of PES support before (left) and after (right) depositing GOP coating. The effective separation length of the hollow fiber membrane is 43 mm; the membrane was sealed in a Pyrex tube at two ends using epoxy, and two holes on each side were drilled into the Pyrex tube. The coated PES support surface shows a uniform dark gray color, suggesting a high-quality coating. **b** Surface and **c** cross-sectional SEM images of a GOP (10 wt.% piperazine) membrane. The scale bars in **b** and **c** are 200 nm and 50 nm, respectively

Ultrathin, uniform GO coating was successfully deposited on the inner surface of a PES hollow fiber support with an average pore size of 25 nm. The digital picture in Fig. 2a shows a GOP hollow fiber membrane housed in a Pyrex tube. The inset picture clearly shows the color difference between the blank PES support and PES with GOP membrane, and the uniform dark gray color after coating suggests a high-quality coating on the inner surface. Before preparing GOP membranes, we deposited GO membranes on a PES hollow fiber to ensure the proposed deposition process indeed resulted in high-quality coating; besides, CO_2_ separation performance of GO membranes can be used as a base for comparison with GOP membranes. Field emission scanning electron microscope (FESEM) images (Supplementary Fig. 3a and 3b) shows the surface morphology of the PES hollow fiber support before and after GO coating with the developed seeding and coating method. Clearly, the pristine pores of the PES hollow fiber support have been fully covered by GO flakes, and no obvious holes or cracks can be observed. The cross-sectional view of a GO-coated membrane (Supplementary Fig. 3c) shows an average GO coating thickness of 9 nm after 1-min seeding and 10-min coating using 0.1 mg ml^−1^ of GO solution. The surface of a GO membrane without the seeding step is shown in Supplementary Fig. 4. Compared to that the one with the seeding step, a very rough coating layer can be seen, indicating the importance of the seeding step.

A GOP membrane was deposited following the same deposition condition but using a solution with GO concentration of 0.1 mg ml^−1^ and 10 wt.% piperazine. The SEM image in Fig. 2b reveals the surface of the hollow fiber support was uniformly coated with a GOP layer. The cross-section of the GOP hollow fiber membrane (Fig. 2c) shows that the thickness of the GOP selective layer is ~16 nm. Thicker GOP coating apparently is attributed to the high content of piperazine loading between the GO sheets (see more detailed discussion below).

**Fig. 3:**
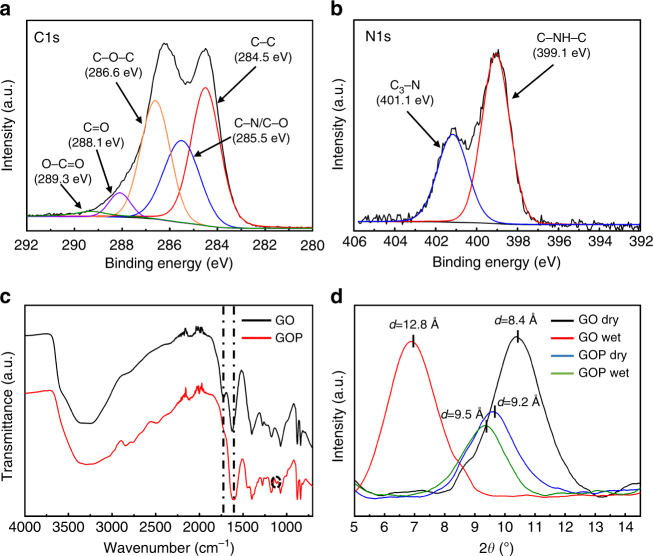
Characterization of surface functional groups of GO and GOP membranes. XPS spectra of GOP membrane in C 1s region (**a**) and N 1s region (**b**); **c** ATR-FTIR spectra of GO and GOP membranes, showing the new carbon-nitrogen peak appeared and the original carboxyl and carbon double bond shifted; **d** XRD patterns of GO and GOP membranes at dry and wet states, respectively, and the corresponding *d*-spacing

To confirm the chemical bonding between the piperazine and the GO sheets, X-ray photoelectron spectroscopy (XPS) analysis was performed for the GO and GOP membranes. The elemental analysis (Supplementary Table 2, and Fig. 5a, b) shows that the O/C ratio decreased from 0.61 (GO membrane) to 0.43 (GOP membrane), suggesting oxygen on SLGO must have been partially removed by the reaction between oxygen-containing groups on SLGO and piperazine^32^. The nitrogen percentage in GO membrane was negligible, whereas the GOP membrane exhibited 9.1% nitrogen (Supplementary Table 2). The C 1s region of GOP membrane was deconvoluted and shown in Fig. 3a. As a base for comparison, the C 1s region of GO membrane (Supplementary Fig. 5c) was also deconvoluted and showed five peaks representing different species with different binding energy as follows: C−C (284.5 eV), C−N/C−O (285.5 eV), C−O−C (286.6 eV), C=O (288.1 eV) and O−C=O (289.3 eV). Compared with GO membrane, GOP membrane showed significantly enhanced C−O/C−N peak, whereas the O−C=O peak almost disappeared and the C−O−C peak showed a significant decline in intensity. The epoxy groups offer sites for the nucleophilic substitution reaction for amines^32, 33^. As a consequence, new covalent chemical bonds (C−N−C) were formed from the ring-opening reaction of the epoxide group with −NH_2_ on piperazine. Another possible reaction might be between carboxyl groups and piperazine amines. Similar results were found in other studies^32–34^. The N 1s region of the GOP membrane was shown in Fig. 3b. No nitrogen peak was found in GO membranes (Supplementary Fig. 5a), but two distinct peaks were observed in the GOP membrane. Piperazine has a single-nitrogen peak from C−NH−C^35^, and it was detected at binding energy of 399.1 eV. The peak at 401.1 eV corresponds to tertiary amine (C_3_−N), indicating chemical bonding between GO and piperazine.

We further verified chemical bonding of piperazine with GO surface in the GOP membrane by attenuated total reflectance-Fourier transform infrared spectroscopy (ATR-FTIR). As shown in Fig. 3c, characteristic absorption peaks of GO membrane were observed at wavenumbers of 3300, 1724, 1624, 1397, 1171, and 1071 cm^−1^, corresponding to stretching of hydroxyl (OH), C=O in carboxyl group, C=C in aromatic ring, C−O in carboxyl group, C−O in epoxy group, and C−O in alkoxy group, respectively. After reaction with piperazine, the peaks of C=O in carboxyl group and aromatic carbon double bond (C=C) shifted and overlapped into a broad peak at 1589 cm^−1^, which also contains the bending of N−H. This could be interpreted as evidence that the carboxyl groups on the GO surfaces react with amine groups of piperazine. In addition, the hydroxyl content obviously decreased, while a new peak at 1110 cm^−1^ emerged, which refers to C−N bonds, confirming the reaction between the amine groups with the epoxy and/or hydroxyl groups of GO.

X-ray diffraction (XRD) analysis of the as-prepared GO and GOP membranes in both dry and wet states were conducted, and their patterns were shown in Fig. 3d. In dry state, the GO membrane showed a *d*-spacing of 8.4 Å, whereas the GOP membrane had a larger *d*-spacing of 9.2 Å, attributing to the intercalated piperazine molecules (both chemically and physically) between GO flakes. Both membranes were then presoaked in deionized (DI) water for 5 h before conducting XRD measurements in the wet state. In the wet state, the *d*-spacing of the GO membrane increased to 12.8 Å, whereas that of GOP membrane changed slightly to 9.5 Å, an ~15 times smaller increase than that of GO membrane. Change in *d*-spacing stems from the water molecules that enter the GO layers, resulting in the destruction of *π*–*π* interactions and thus the stretching of *d*-spacing^36^. However, for the GOP membrane, piperazine is chemically bonded to the surface of GO sheets in three possible states (Supplementary Fig. 6), and with the presence of piperazine bonded with two adjacent GO flakes, the formed C−N covalent bonds are expected to create a strong resistance to the *d*-spacing stretching, leading to much slighter increase^32^.

### Membrane gas permeation and selectivity

Gas permeation through GO and GOP hollow fiber membranes was carried out using a home-made permeation system connected with gas chromatography (GC) for composition analysis (Supplementary Fig. 7). Single gas and mixed gas (CO_2_: N_2_ = 15%: 85% in vol%) permeation was conducted under both dry and wet conditions. As reported from literature^19, 26^, most gas molecules, such as H_2_, N_2_, O_2_, CO_2_, and CH_4_, could pass through the intrinsic nanochannels between the GO flakes with low permeation resistance in dry feed condition. Our GO membranes presented a low CO_2_/N_2_ selectivity of 1.29 under dry condition, obviously due to the relatively large interlayer nanochannels (refer to Fig. 3d). Note that the real flue gases are usually saturated with water vapor. We then tested our GO membrane with water vapor in the feed (wet feed condition). We found that all the gas permeances decreased in wet feed condition due to the transport resistance generated from the adsorbed water molecules between the GO flakes. Apparently, adsorbed water molecules significantly hinder the gas permeation through GO membranes. Yet, gases have different solubility and diffusivity in water. At room temperature, gas solubility coefficients in water follow the sequence of CO_2_ > O_2_ > CH_4_ > N_2_ > H_2_, and gas diffusion coefficients in water follow the reversed order of H_2_ > N_2_ > CH_4_ > O_2_ > CO_2_. Kim et al.^26^. reported that under high humidity, contribution of solubility to gas permeance was greater than that of diffusion coefficient, and CO_2_ permeated through the GO membrane faster than other gases based on the solution-diffusion mechanism. Therefore, the presence of water in GO membranes would result in higher CO_2_ permeance than that of other gases, and increase selectivity of CO_2_ over other gasses. In our study, the ideal CO_2_/N_2_ selectivity (ratio of permeances of pure CO_2_ and N_2_) of GO membrane was 50 at 25 °C, much higher than the Knudsen selectivity (0.799)^37^, demonstrating the good separation capability of GO hollow fiber membranes. The CO_2_ permeance at 25 °C, however, was only 49 GPU, although GO coating was already as thin as 9 nm. This is comparable to Kim et al.’s result of ultrathin GO coating (<5 nm) on flat sheet PES support deposited by spin coating^26^, demonstrating high quality of our GO coating. In mixed gas permeation measurements, GO membrane showed a CO_2_/N_2_ selectivity of 34 and CO_2_ permeance of 35 GPU, slightly lower than ideal selectivity and pure CO_2_ permeance. As shown in Supplementary Fig. 8, with the increase of the permeation temperature in the mixed gas separation, CO_2_ permeance exhibited very small change whereas N_2_ permeance increased four times, resulting in an obvious decrease of selectivity from 34 at 25 °C to ~12 at 80 °C. This may be because the solubility decrease and diffusivity increase for CO_2_ are well balanced, while N_2_ diffusivity dominates the increase for the permeance at higher temperature.

**Fig. 4:**
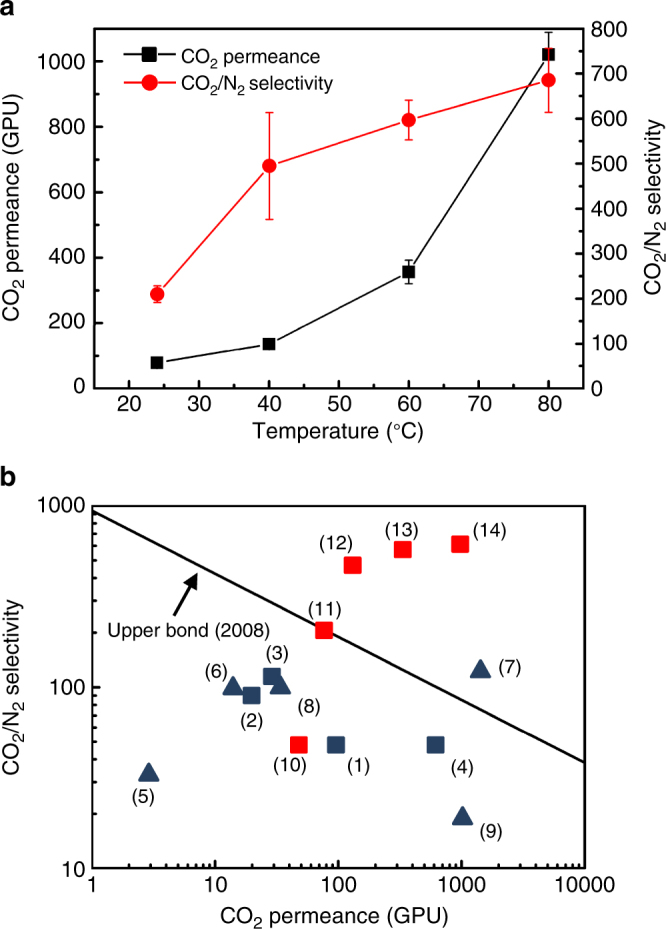
CO_2_/N_2_ mixture separation performance of GOP membranes and comparison with membranes reported in the literature. **a** Influence of temperature on GOP membrane performance for mixed gas (CO_2_: N_2_ = 15%: 85%, vol%) separation under wet condition. It is noted that some error bars of CO_2_ permeance were smaller than the cubic symbol, and thus they are not shown in the figure. **b** Performance comparison of GOP membranes with polymeric membranes (black line) reported in 2008 upper bound^11^ (modified upper bond (in GPU), thickness of polymer membranes is assumed to be 100 nm) and some representative recent membranes^6–8, 19, 21–23, 41, 42^. Squares denote the GO-based membranes; triangles denote the polymer-based membranes. (1) GO^19^. (2) GO-Pebax^22^. (3) GO-PEI^23^. (4) GO-Borate^21^. (5) Pebax-PEG-POSS^6^. (6) PEI-Silica-Pebax^7^. (7) PDMS^41^. (8) PVAm^42^. (9) PEG-based^8^. (10) GO on single feed tests, this work. (11) to (14) GOP at 24, 40, 60, 80 °C, respectively, wet mixed feed, this work

To further increase CO_2_ permeance, CO_2_-philic agent, piperazine, was incorporated into GO membranes to form grafted piperazine brush for facilitated transport. We found GOP membrane prepared using 10% piperazine in the coating solution showed the best performance (Supplementary Table 3), and thus focused our discussion below on this GOP membrane. Effect of temperature on CO_2_ permeance and CO_2_/N_2_ selectivity was shown in Fig. 4a, and CO_2_ permeation activation energy was also examined as shown in Supplementary Fig. 9. CO_2_ permeance increased exponentially with the increase of temperature and followed an Arrhenius relationship, suggesting activated diffusion of CO_2_ in the GOP membrane^38, 39^. The CO_2_/N_2_ selectivity reached higher than 500 at temperature above 40 °C. This is because the CO_2_ permeance increase, caused by the facilitated transport through GOP nanochannels at high temperature, is much greater than the increase of N_2_ permeance caused by higher diffusion rate. In our mixed CO_2_/N_2_ gas separation test, the GOP membrane showed CO_2_/N_2_ selectivity as high as 680 and CO_2_ permeance of 1020 GPU under wet condition at 80 °C, exhibiting the best performance ever compared with all the reported GO-based membranes. Lower relative humidity at 90% was also examined, and negligible influence on the separation performance was found. Compared to our GO membrane, the GOP membrane had twenty times higher CO_2_ permeance as well as more than 10 times higher CO_2_/N_2_ selectivity at 80 °C in wet feed condition, attributed to the fast and selective nanochannels brushed and alternated with CO_2_-philic agent, piperazine. Under dry condition, GOP membranes exhibited a CO_2_ permeance of 1890 GPU and an N_2_ permeance of 1870 GPU, indicating that the water in the GOP membrane almost completely hinders the transport of N_2_ but generates a much lower resistance for CO_2_ permeation.

In this layered GOP hollow fiber membrane, both the solution-diffusion and facilitated transport mechanism occur concurrently. Both selectivity and permeance are expected to be greatly enhanced by introducing the amines into the GO nanochannels, resulting from the reversible reactions between CO_2_ and amine groups as follows:

$$2{\mathrm{CO}}_2{\mathrm{ + 2}}{\rm RR}^\prime {\mathrm{NH}} + {\rm H_2O} \leftrightharpoons {\rm RR}^\prime {\mathrm{NCOOH}} + {\rm RR}^\prime {\rm NH_2^ +} + {\mathrm{HCO}}_3^ -$$

$${\mathrm{CO}}_2{\mathrm{ + R}}{\rm R}^\prime {\rm R}^{\prime \prime }{\mathrm{N + }}{\rm H_2O} \leftrightharpoons {\rm RR}^\prime {\rm R}^{\prime \prime} {\rm NH}^ + + {\mathrm{HCO}}_3^ -$$

Piperazine would be prone to form zwitterion first and then generate carbamate or bicarbonate in the presence of water^2, 40^. Detailed CO_2_ facilitated transport mechanism was illustrated in Supplementary Fig. 10. The bonded amine groups offer tertiary (RR′R″N) amines, which act as weak base catalysts for the CO_2_ hydration reaction in the presence of water^40^. The hydrogen bonding between amine and water weakens the H−O bond and enhances the nucleophilic reactivity of water toward CO_2_, leading to the bicarbonate formation^40^.

Overall, the amine brushes catalyze the HCO_3_^−^ conversion from CO_2_, offering a rapid diffusion of CO_2_ in the hydrated GO nanochannels. During a 1200-min testing, the GOP membrane displayed good permeance stability (<20% variation) with negligible change of selectivity. The separation performance of our GOP membrane was compared with the Robeson’s upper bound^11^ and others’ work in the same field^6–8, 19, 21–23, 41, 42^, as illustrated in Fig. 4b. It should be noted that Fig. 4b is a modified upper bound figure in which permeance (in GPU; membrane thickness is assumed 100 nm), instead of permeability, was used, because the permeance is a more practical membrane property than permeability in membrane separation application. It is clearly shown that our results stand out much better in terms of CO_2_ permeance and CO_2_/N_2_ selectivity.

In summary, we developed a facile and potentially scalable method to prepare layered GO membranes brushed and alternated with a CO_2_-philic agent on the inner surface of a hollow fiber via a two-step modified vacuum filtration deposition process. With the facilitated transport of CO_2_ molecules between the GO nanochannels, the as-prepared GOP hollow fiber membranes showed extremely high CO_2_ permeance and superior CO_2_/N_2_ mixture selectivity at 80 °C under simulated flue gas feed condition. The grafted amine-brushes in the GO nanochannels offer reaction sites with CO_2_ molecules, and thus considerably improve CO_2_/N_2_ selectivity and CO_2_ permeance. Considering the high area to volume ratio of hollow fibers, facile GO-based coating deposition process and highly efficient CO_2_/N_2_ separation performance under simulated flue gas condition, the ultrathin, layered GO hollow fiber membranes with amine-brushes exhibit a great potential for CO_2_ capture from flue gas.

## Methods

### Graphene oxide synthesis and membrane preparation

Graphene oxide was synthesized by a modified Hummers method. Ultrathin GO membranes were fabricated by GO seeding and vacuum-assisted coating method with our laboratory-designed coating system (Supplementary Fig. 1). The detailed preparation procedures are presented in Supplementary Methods. The morphology and microstructure of the samples were investigated by FE-SEM (Zeiss Ultraplus Thermal), XPS (Zeiss Ultraplus Thermal), ATR-FTIR (PerkinElmer Spectrum 100), and XRD (Rigaku MiniFlex II) examinations.

### Scanning electron microscopy

The membrane surface and cross-sectional morphology were observed by Field Emission Scanning Electron Microscope (Zeiss Ultraplus Thermal FESEM). The sample was cryogenically fractured in liquid nitrogen, and sputtered for 60 s to obtain a thin gold layer before SEM tests. The uniformity of coating thickness was confirmed from FESEM (see Supplementary Fig. 11).

### X-ray photoelectron spectroscopy

The membrane chemical composition was analyzed by X-ray photoelectron spectroscopy (Kratos Axis Ultra DLD XPS system). Samples were prepared with the same coating conditions, followed by four times thorough wash with DI water on the membrane surfaces, 1-h heat treatment at 80 °C (for GOP membrane) and then soaked in water for another 20 h to remove residual chemicals.

### Attenuated total reflectance-Fourier transform infrared spectroscopy and X-ray diffraction

The membrane chemical properties were characterized by attenuated total reflectance-Fourier transform infrared spectroscopy (PerkinElmer Spectrum 100 FT-IR). Membranes in dry and wet states were investigated by wide-angle X-ray diffraction (Rigaku MiniFlex II XRD) to measure the *d*-spacing of GO and GOP layers. Dry samples were vacuum dried in oven at 40 °C overnight, while the wet samples were pre-soaked in DI water for 5 h to reach the hydrated state.

### Gas permeation

The measurements of membrane transport properties were conducted by using a gas permeation system (Supplementary Fig. 7). The membrane coating module was loaded in a cylindrical stainless-steel cell with an active membrane area of 1.35 cm^2^ inside an oven (Forced Convention Oven, JeioTech Co., Ltd. US) with accurate temperature control. The feed and the sweep gas flows were designed to be perpendicular in the cell. Feed gas consists of CO_2_ (99.999%, Praxair, US) and N_2_ (99.999%, Praxair, US) in different concentration by adjusting their flow rate with mass flow controller (MFC). Helium (99.999%, Praxair, US) as sweep gas, carried the permeance gas for composition analysis. Before contacting the as-coated hollow fiber membranes, the feed gas and the sweep gas (He) were saturated with water vapor by bubbling through the humidifiers and then passing an empty bottle to remove the condensate water. The relative humidity (RH) at the feed side was detected by a humidity sensor and was >99%. After the membrane separation process, both the retentate and permeate streams leaving the oven were cooled to ambient temperature in their respective water knockout vessels to remove the condensed water before the streams entered an Agilent HP-5890-II gas chromatograph (Agilent Technologies, Palo Alto, CA) for gas composition analyses. Helium was used as the carrier gases for the TCD detector in GC. The gas chromatograph column used was of the RESTEK Rt-U-BOND 19750 (Restek, Bellefonte, PA). Each of the membrane permeation measurement was taken after the membrane had been exposed to the feed and permeate streams under a specific condition (temperature and pressure) until the GC shows constant result for at least 30 mins, which allowed for steady-state permeation. The permeance of CO_2_ and N_2_ was calculated from the sweep gas flow rate and its composition. The permeate side pressure in the system was maintained at the atmosphere pressure.

### Data availability

All the data supporting the results in this study are available from the authors.

## Electronic supplementary material

Supplementary Information
