# Supplementary material for: Ultrathin graphene oxide-based hollow fiber membranes with brush-like CO2-philic agent for highly efficient CO2 capture
Source: Nat Commun. 2017 Dec 13;8:2107. doi: 10.1038/s41467-017-02318-1 (PMC5727382; doi:10.1038/s41467-017-02318-1)
Supplement: Supplementary file 1 — Supplementary Information [file 41467_2017_2318_MOESM1_ESM.pdf]

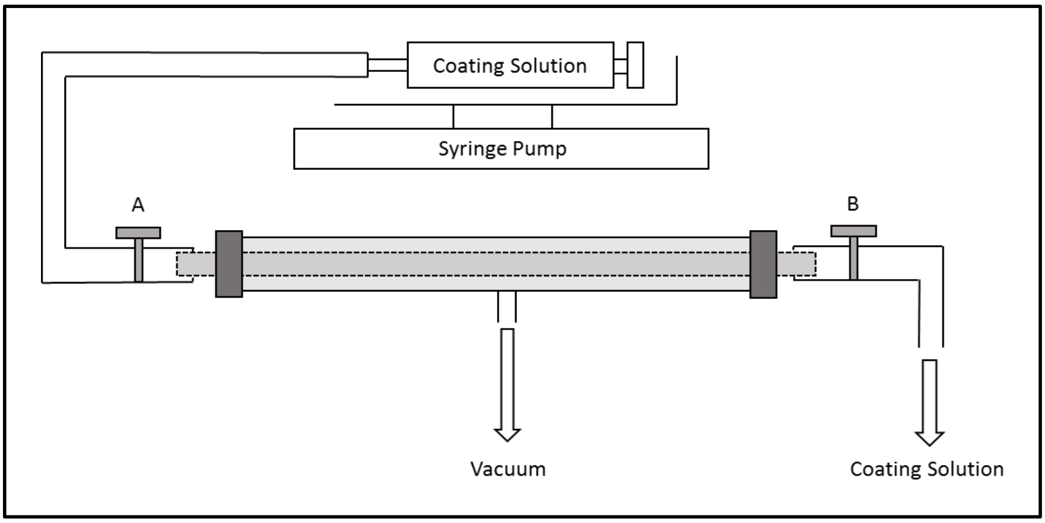

Supplementary Figure 1. Hollow fiber membrane coating setup. The polyethersulfone (PES) hollow fiber support was installed and fixed into the cylindrical module before the vacuum filtration coating. Coating solution was injected into the PES support with a constant flow rate.

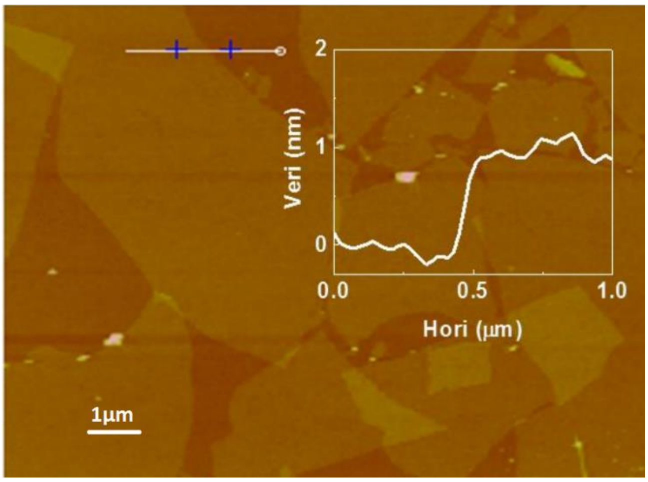

Supplementary Figure 2. AFM image of GO flakes, showing the single-layered graphene oxide (SLGO) with thickness of about 1 nm.

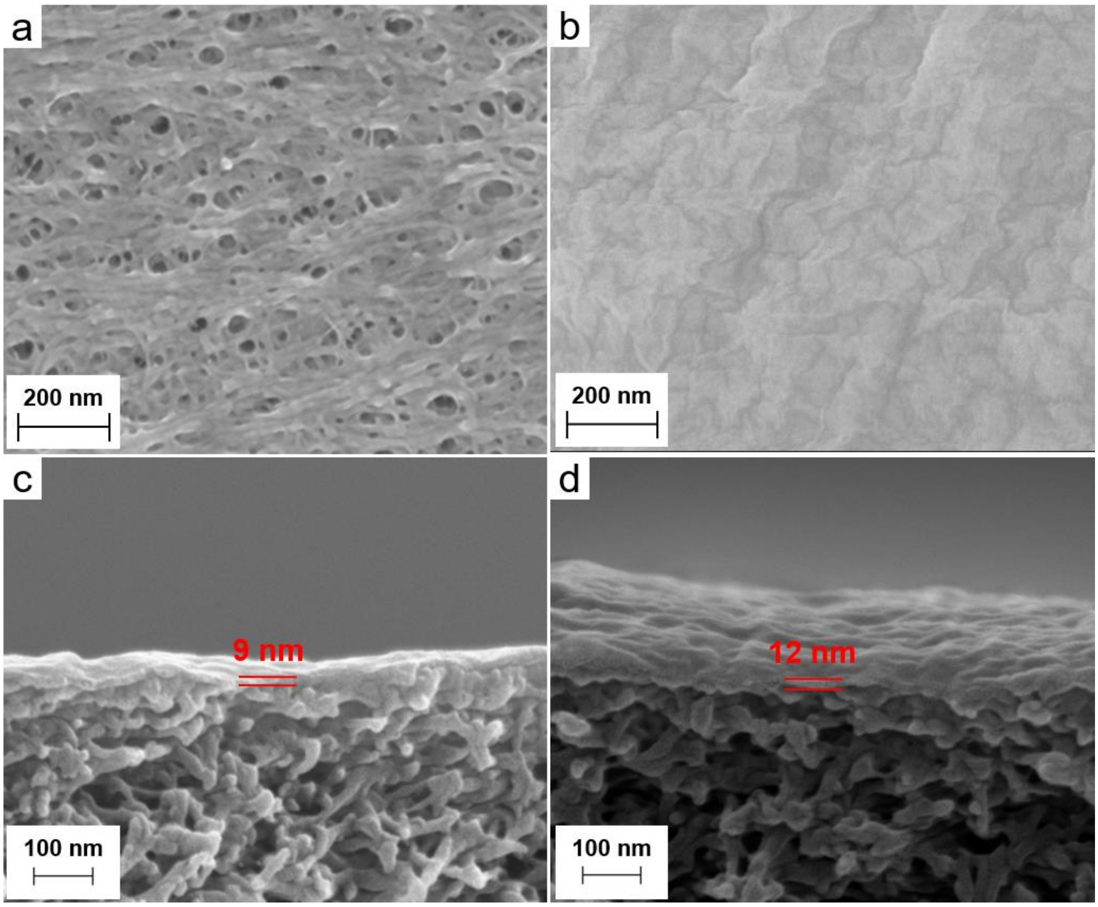

Supplementary Figure 3. (a) Surface SEM image of PES hollow fiber support. (b) Surface SEM image of GO hollow fiber membrane. (c) Cross-sectional SEM image of GO hollow fiber membrane, exhibiting the active layer thickness of 9 nm. (d) GOP hollow fiber membrane with 1 wt.% piperazine in coating solution, exhibiting the active layer thickness of 12 nm. Same coating steps with GO membranes: 1 min for GO seeding, 10 min for vacuum coating and drying. The cross-sectional image of GOP hollow fiber membrane with 10 wt.% piperazine in coating solution (Fig. 2c) has thickness of 16 nm. This shows the active layer thickness increased with the increase of the piperazine concentration. 10 wt.% piperazine-GO coating solution provides more than two-times higher piperazine loading (from 9 nm to 16 nm) in the GO nanochannels than that with 1 wt.% piperazine-GO coating solution (from 9 nm to 12 nm).

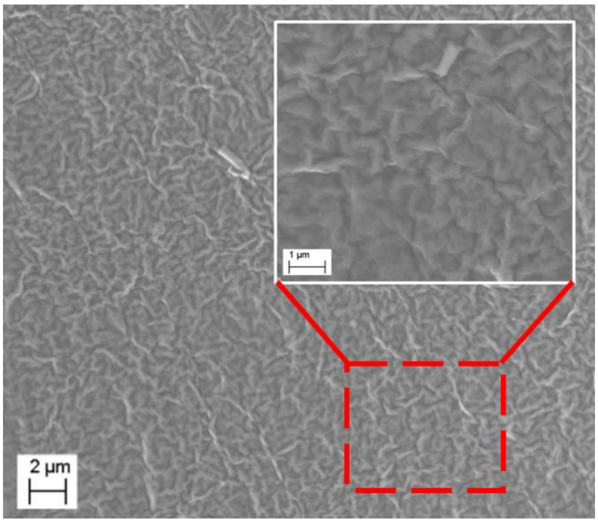

Supplementary Figure 4. GO hollow fiber membrane coated without seeding step. Extremely rough coating surface was observed, indicating the importance of seeding step.

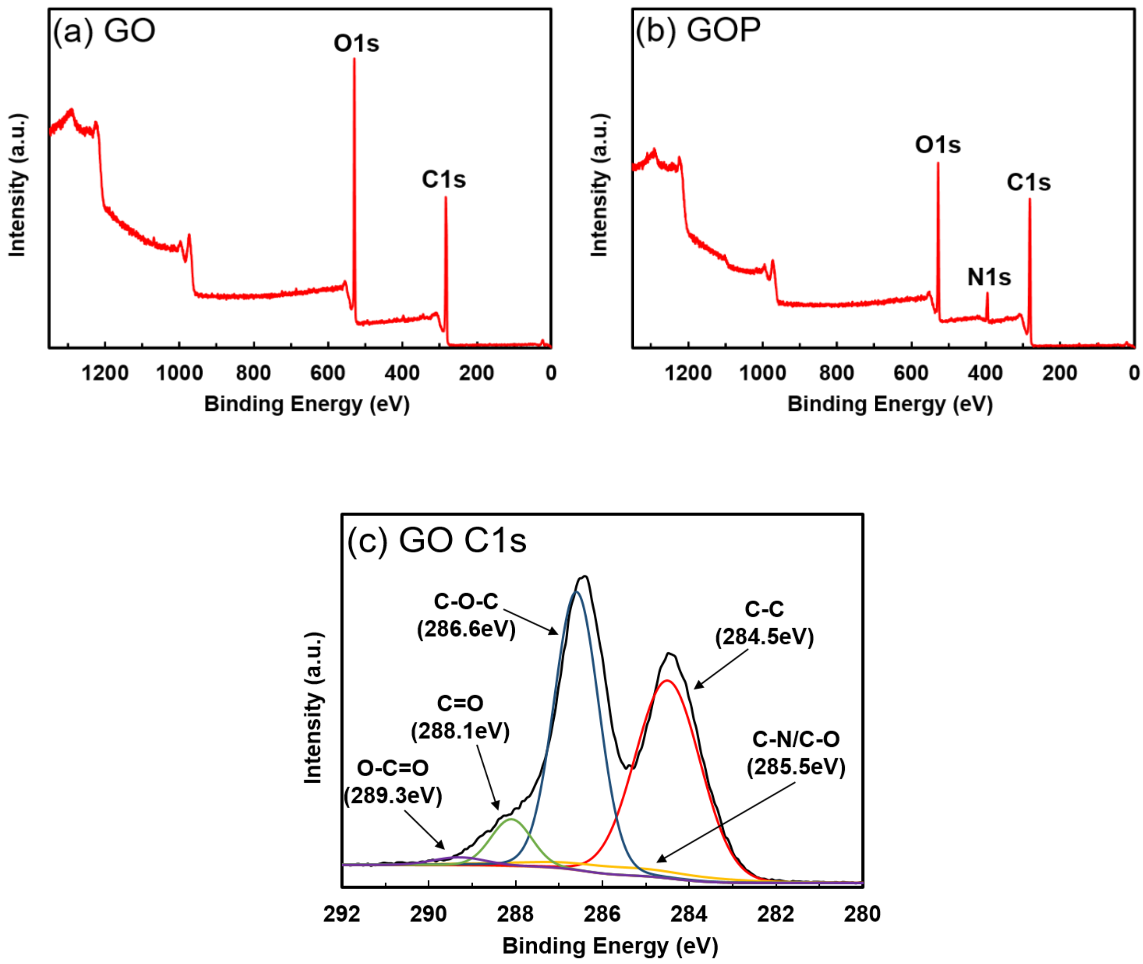

Supplementary Figure 5. (a) and (b) XPS elemental analysis of GO membranes and GOP membranes, respectively. Membranes are washed with deionized water for 20 hours to remove the physically adsorbed piperazine. The oxygen content significantly decreased, and the nitrogen peak emerged in GOP membranes, indicating the oxygen-containing groups in GO surfaces were consumed by the amine crosslinking reaction. (c) C 1s region fitted analysis of GO membranes. Five peaks represent different carbon species as shown in the figure.

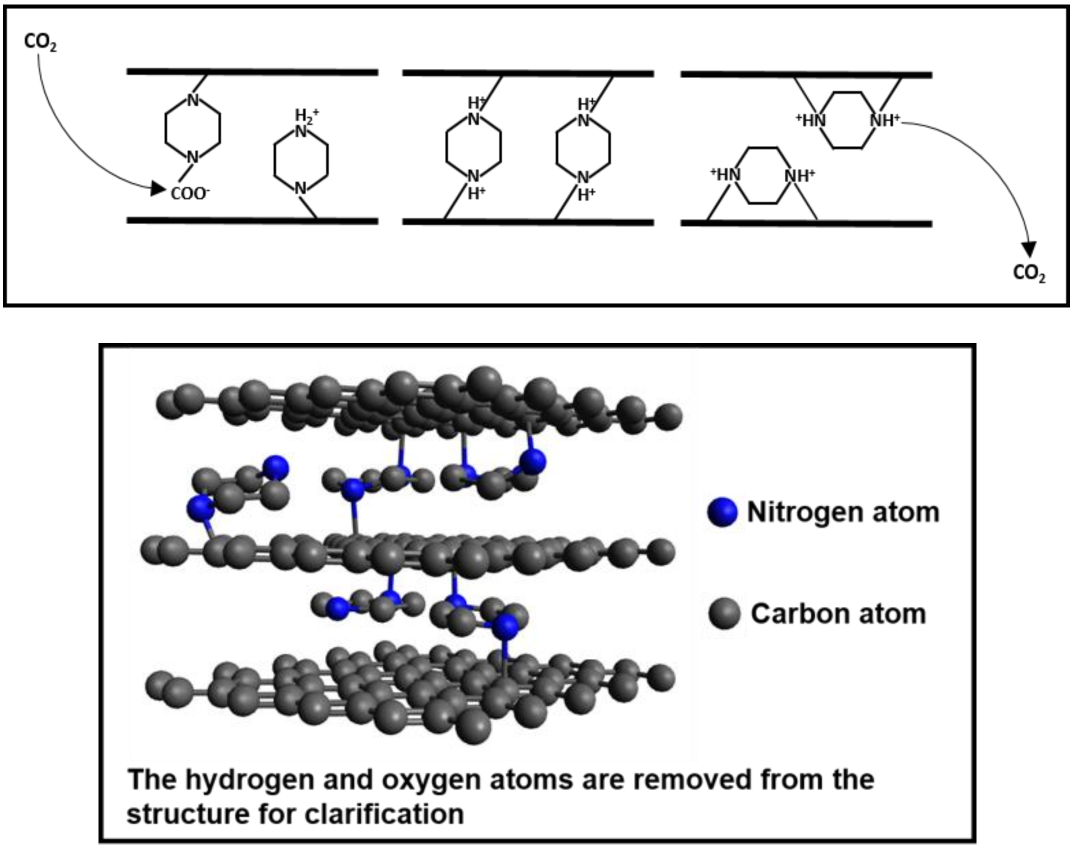

Supplementary Figure 6. Structure diagram of three possible different chemically bonded piperazine CO_2 carriers between GO layers. CO_2 molecules enter the GO nanochannels and reversibly reactive with the amine groups in presence of water to form carbamate or bicarbonate. The chemically bonded amine molecules act as fixed and fast transport carriers of CO_2.

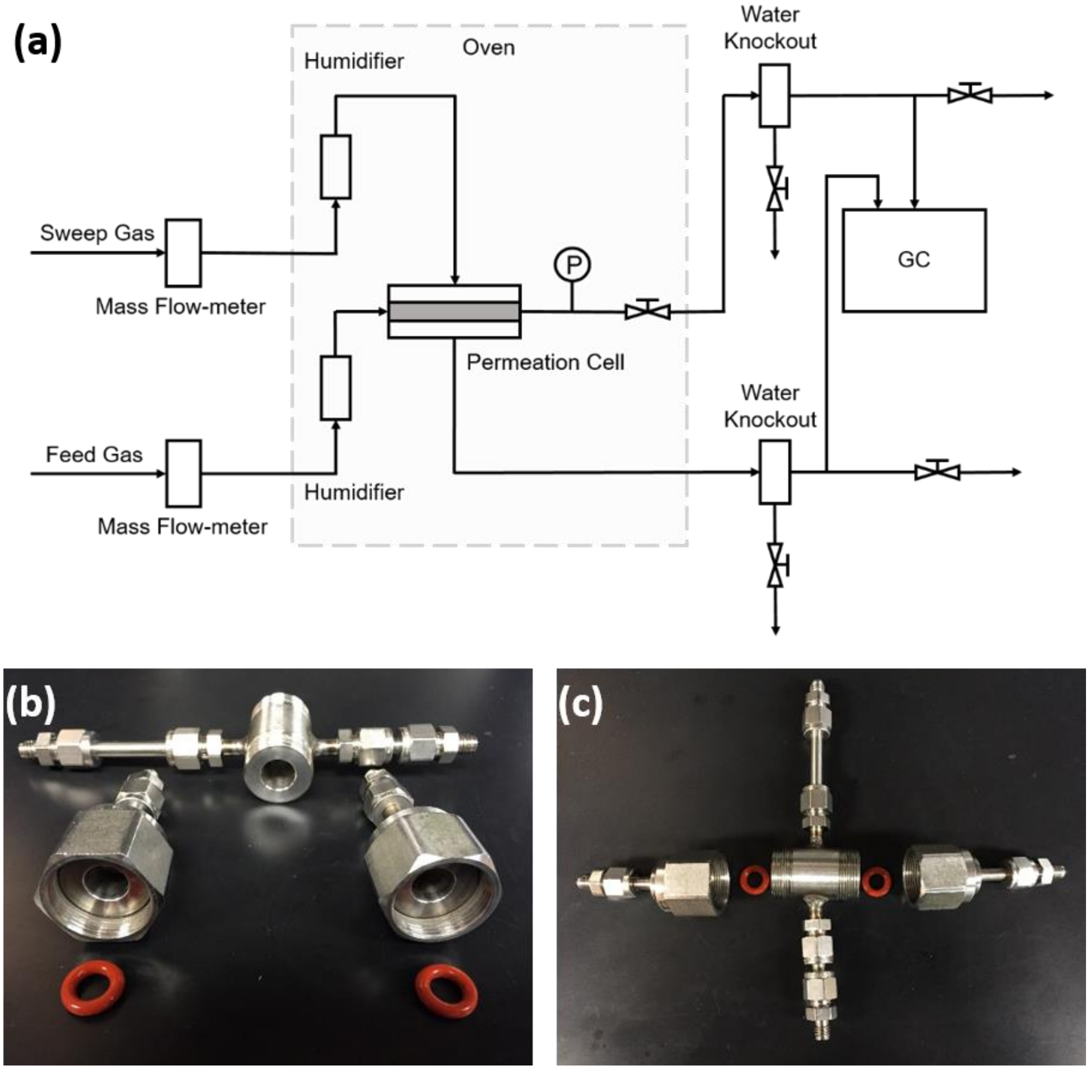

Supplementary Figure 7. (a) Schematic diagram of gas permeation system. (b) and (c) Photographs of hollow fiber permeation cell.

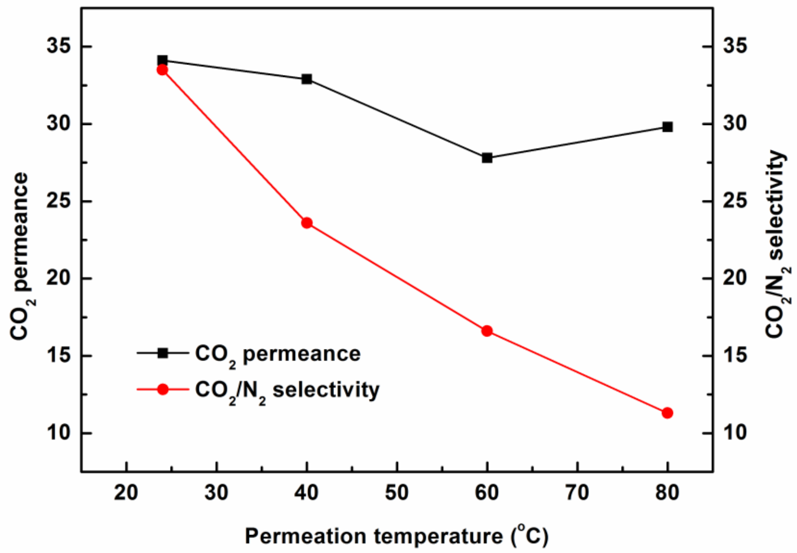

Supplementary Figure 8. Influence of temperature on GO membrane performance with wet mixed feed gas (CO_2: N_2=15%: 85%, vol%). The CO_2 permeance changed very slightly with temperature increase, indicating there is no absorption/diffusion mechanism on GO membrane gas separation. The CO_2/N_2 selectivity significantly decreased with temperature increase, attributing to the relatively faster gas molecules transport between the GO nanochannels caused by higher diffusion rate at high temperature. For CO_2, the solubility decrease and diffusivity increase reached a good balance, resulting in a negligible change of permeance; while for N_2, the increased diffusivity dominates the permeance increase at higher temperature due to the extremely low solubility of N_2 in water.

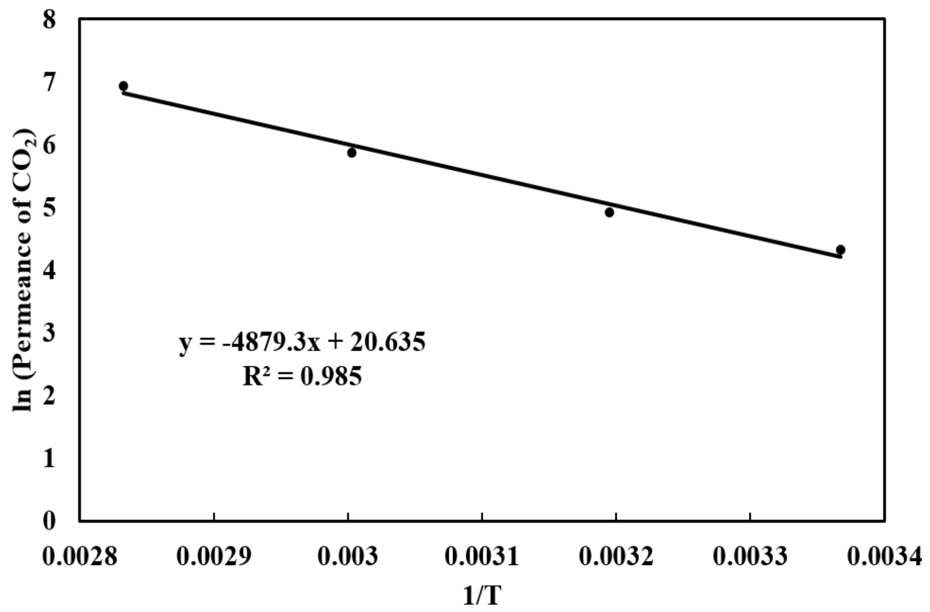

Supplementary Figure 9. The $lnP_{CO_2} - 1/T$ plot for CO_2 absorption/diffusion activation energy of GOP membranes. The activation energy of CO_2 permeation in GOP membranes can be calculated from Arrhenius equation,

$$P_{CO_2}(T) = A_0 \times \exp(\frac{-E_a}{RT})$$

where P_{CO_2} is the CO_2 permeance of GOP membranes, A_0 is the Arrhenius pre-exponential factor, E_a is the activation energy associated with diffusion rate and reaction rates (forward and backward), R is the universal ideal gas constant, and T is the absolute temperature. The plot revealed that the activation energy is 40.569 kJ/mol, indicating the facilitated CO_2 transport stemed from the high absorption/diffusion rate.

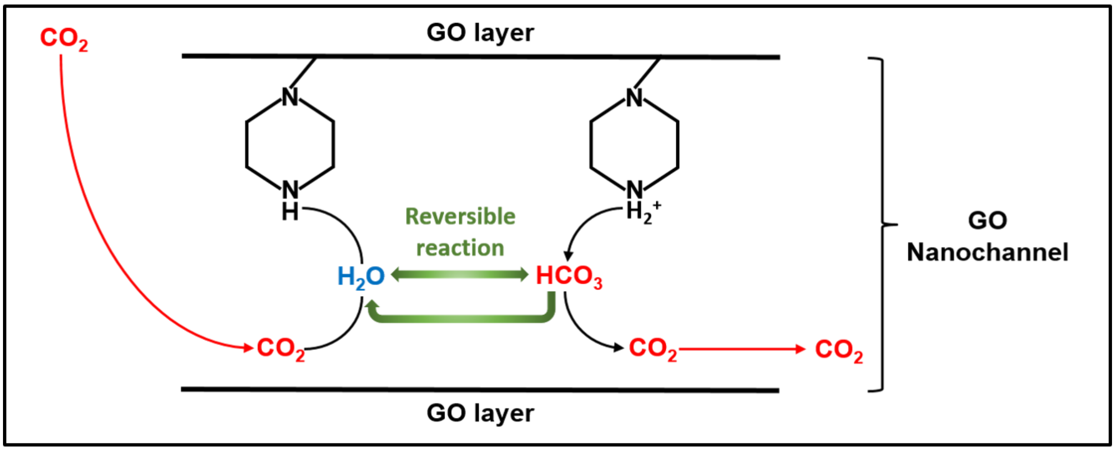

Supplementary Figure 10. The CO_2 facilitated transport mechanism. Secondary amine groups used as example. CO_2 molecules dissolve in GO membrane layer at the feed side, then react with amine groups (both secondary and tertiary amines) and water to form HCO_3^-, which diffuses through the GO nanochannels and layers much faster than CO_2 molecules, and then dissociates into CO_2 and water at the permeate side.

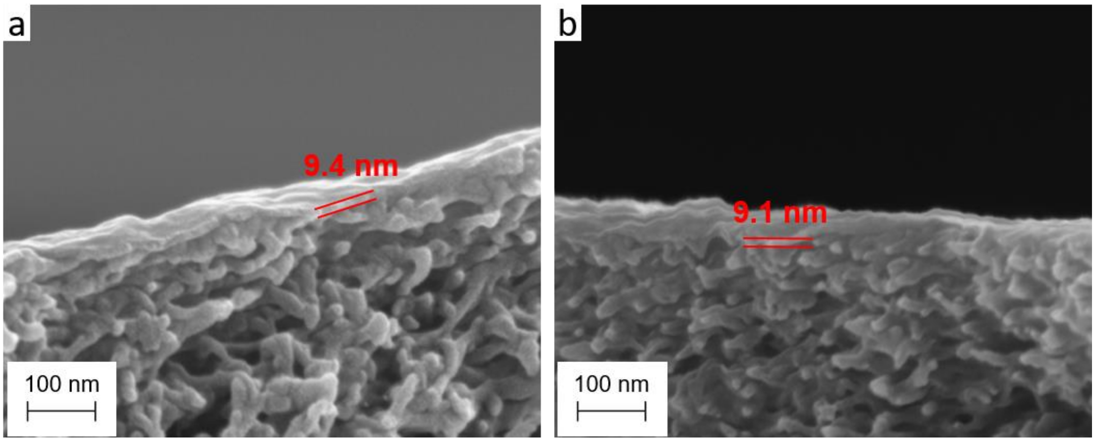

Supplementary Figure 11. Cross-sectional SEM images of the end part and the central part of GO membrane. The end part (a) coating exhibits a thickness of 9.4 nm and the central part (b) coating exhibits a thickness of 9.1 nm, illustrating the uniform coating thickness at every place of the hollow fiber inner surface.

Supplementary Table. 1 Non-selective GO membrane preparation methods

**Preparation method**	**CO_2 permeance in (GPU)**	**Selectivity (CO_2/N_2)**
Flow coating	3,477±241	1.17±0.02
Filling-sealing-vacuum filtration	2,585±150	1.13±0.10

Supplementary Table 2. XPS Elemental Analyses of Composite GO and GOP Membranes. The nitrogen content increased to 9.06 % in GOP membranes, and the O/C content ratio decrease from 0.61 in GO membranes to 0.43 in GOP membranes.

**Sample**	**C %**	**N %**	**O %**	**O/C**
GO	61.23	–	37.53	0.61
GOP	63.57	9.06	27.37	0.43

Supplementary Table 3. Thickness, CO_2 permeance and selectivity of GOP membranes prepared by different piperazine concentrations in coating solution. Different thickness demonstrated the different piperazine loading with the change of piperazine concentration in coating solution. Higher loading exhibited higher CO_2 permeance, probably resulting from the higher amine grafting density in the GO nanochannels.

**Piperazine concentration in coating solution, wt%**	**GOP membrane thickness, nm**	**CO_2 permeance, GPU**	**Selectivity (CO_2/N_2)**
1	12	220	190
5	15.1	770	430
10	16	1,020	680

Supplementary Methods

Materials

Expandable graphite (EAG, Grade 1721) was purchased from Asbury Carbon Inc. (NJ, US). Concentrated sulfuric acid (H_2SO_4, 98 wt.%) and hydrogen peroxide aqueous solution (H_2O_2, 30 wt.%) were purchased from Sigma-Aldrich Corp. (St. Louis, MO, US). Hydrochloric acid (HCl) and potassium permanganate ($KMnO_4$) were purchased from Fisher Scientific Inc. (Pittsburgh, PA). Piperazine (99%; molar mass: 86.136 g/mol; melting point: 106 ^oC; boiling point: 146 ^oC) was purchased from Sigma-Aldrich Corp. (St. Louis, MO, US). All the reagents were of analytical grade and used without further purification. Deionized water processed through a PURELAB Flex system (ELGA LabWater, Woodridge, IL, US) was used throughout. Microporous polyethersulfone (PES) hollow fiber membranes (ID 1.0 mm, OD 1.4 mm, average pore size: 0.025 μm) were purchased from Hydranautics, Inc. (Oceanside, CA, US).

Membrane preparation and coating setup

Coating solution was prepared by diluting GO suspension to specific concentration (0.1 mg/mL in this work) and then sonicated for 10 mins. For GO-Piperazine (GOP) membrane, piperazine was then added into the GO dispersion and sonicated for 15 mins to form a homogeneous coating solution.

The coating was conducted with different methods to achieve selective GO membrane. First, flow coating method was examined at different flow rate of coating solution and different drying steps, but the membrane exhibited no CO_2/N_2 selectivity possibly due to the hydrophobic surface of PES supports. Filling-sealing-vacuum drying method was then investigated. Hollow fiber was firstly filled with GO coating solution (0.1 mg/mL), and two ends were sealed, then the hollow fiber was dried in vacuum oven to remove all the DI water from the coating solution at 40 ^oC for overnight. Yet the membrane also showed no CO_2/N_2

selectivity probably due to the non-uniform GO coating layer. CO_2 permeance and selectivity of non-selective membranes prepared by flow coating and filling-sealing vacuum drying methods were listed in Supplementary Table 1. Therefore, we then proposed a GO seeding and vacuum-assisted coating method with our home-designed system as demonstrated in Supplementary Fig. 1.

The PES hollow fiber support was firstly fixed into a clear Pyrex tube (55 mm) with high temperature epoxy (Devcon, US) as shown in Fig. 2, then was ready to use for coating. It is noted that the Pyrex tube was drilled to make two holes on the surface at middle position on both sides, allowing sweep gas to flow into the tube and to carry the permeance gas out.

As illustrated in Supplementary Fig. 1, the home-designed coating system involved a syringe to hold coating solutions, a syringe pump (New Era Pump Systems, Inc. US) which can control the flow rate of coating solution, several connecting tubes, a hollow fiber module which fixed a hollow fiber membrane (coating length: 43 mm) and two ends of the module are sealed with epoxy, and a vacuum pump connected to a middle hole of the hollow fiber module (another hole was temporarily sealed by few layer parafilm).

Ultrathin GO membranes were fabricated by GO seeding and modified vacuum-assisted coating method. First, the GO coating solution (0.1 mg/mL) was pumped into the PES hollow fiber membrane by syringe pump with a constant flow rate of 0.5 mL/min until the solution continuously flow through the coating solution exit channel without any air bubble. Subsequently, the vacuum was applied and the GO seeding immediately started. Seeding was stopped after 1 min vacuum filtration time (in this work). To protect the as-seeded GO membrane layers, we simply sealed the two ends of hollow fiber supports with an impulse sealer (PFS-100) and disconnected the coating module from the solution tubes. A vacuum coating and drying step with 10 min was then conducted to pull out all the water from the inside of hollow fiber, and to form a high quality and uniform GO coating layer. Two ends of the as-

coated GO hollow fiber membrane were cut after drying and the membrane was directly used for permeance tests. The uniformity of coating thickness at a whole hollow fiber was confirmed by SEM as shown in Supplementary Fig. 11. Four samples of each type of membrane were prepared and tested for experimental reproducibility.

Gas permeation measurements

The permeation temperature was controlled by the oven, the gas flow rate was calibrated with gas flowmeter (ADM2000, Agilent Tech., DE, US.), and the pressure was adjusted by a back-pressure regulator and measured by a pressure gauge at the exit of feed gas. The feed pressure was set at 1 psi to avoid back-flow of sweep gas, and the sweep pressure was set close to atmospheric pressure. Gas flow rates were accurately controlled by flow meters (Brooks Instrument, Hatfield, PA) at 80 cm^3/min for the feed side and 20 cm^3/min for the sweep side. Feed and sweep gases directly entered the cell as dry gas or passed through the humidifier as wet gas with >99% humidity (measured by SRH77A Temperature/Humidity Instrument, Cooper-Atkins, CT, US.).

Gas permeance of the membranes is customarily expressed in GPU ($1GPU = 1 \times 10^{-6}\ cm^3(STP)/cm^2 \cdot s \cdot cmHg = 3.348 \times 10^{-10}\ mol/(m^2 \cdot s \cdot Pa)$), and is calculated by the equation:[1]

$$(P_i/l) = \frac{J_i}{\Delta P_i A}$$

Where (P_i/l) denotes the gas permeance of "i"; J_i denotes the gas molar flow rate through the membrane (mol/s); ΔP_i denotes the gas partial pressure difference between feed and permeate sides (Pa); A denotes the membrane active area (m^2). The gas selectivity (α_{ij}) was calculated by the equation:

$$\alpha_{ij} = (P_i/l)/(P_j/l)$$

The detecting limit of GC is around 0.02% for measured gases in our experiments, and the CO_2/N_2 selectivity reported in this work is based on the highest detectable N_2 permeance during the mixed gas permeation tests; the actual selectivity is expected to be higher than the reported value.

Supplementary References

1 Madden, W. C. The performance of hollow fiber gas separation membranes in the presence of an aggressive feed stream. (2005).
